# Radiotherapy alone with curative intent in a case of limited-stage extranodal NK/T-cell lymphoma nasal type: a case report and review of the literature

**DOI:** 10.1007/s00277-025-06260-x

**Published:** 2025-04-03

**Authors:** Biancamaria Mandelli, Donatella Caivano, Antonella Fontana, Natalia Cenfra, Sergio Mecarocci, Maristella Marrocco, David Fanciullo, Roberta Mazzarella, Martina Lorenzon, Giada Pacitto, Alessandro Pulsoni

**Affiliations:** 1 Hematology Unit, Department of Translational and Precision Medicine, Policlinico Umberto I, Sapienza University, Rome, Italy; 2 Department of Oncology Radiation Therapy, San Giovanni Addolorata Hospital, Rome, Italy; 3 PhD School in Traslational Medicine and Oncology, Department of Medical and Surgical Sciences and Translational Medicine, Sapienza University, Rome, Italy; 4 Department of Oncology Radiation Therapy, S.M. Goretti Hospital, Latina, Italy; 5 Department of Hematology, S.M. Goretti Hospital, Polo Universitario Pontino, Sapienza Univesity, Latina, Italy; 6 Medical Physics Unit, S.M. Goretti Hospital, Latina, Italy

**Keywords:** Extranodal NK/T-cell lymphoma, Nasal type, Epstein-Barr virus, Radiotherapy, Volumetric modulated arc therapy, Early diagnosis, Personalized treatment

## Abstract

Extranodal NK/T-cell lymphoma, nasal type (ENKTCL-NT), is an aggressive malignancy primarily affecting the sinonasal region, with a strong association with Epstein-Barr virus (EBV) infection. The disease is significantly more prevalent in Asian and Latin American populations. Diagnosis is particularly challenging in nonendemic regions. We present the case of a 78-year-old male with a one-year history of nasal lesions, later diagnosed with ENKTCL-NT. The patient was treated with curative-intent radiotherapy, achieving a complete clinical response. Radiation therapy, particularly utilizing advanced techniques such as Volumetric Modulated Arc Therapy (VMAT), resulted in favorable outcomes with minimal toxicity. This case emphasizes the importance of early diagnosis, accurate staging, and personalized radiotherapy in the management of ENKTCL-NT. Ongoing research into the molecular pathogenesis, treatment strategies, and prognostic factors is crucial for improving outcomes, particularly in advanced-stage disease.

## Introduction

Extranodal NK/T-cell lymphoma, nasal type (ENKTCL-NT), is an aggressive sinonasal malignancy accounting for 12–15% of head and neck cancers, following squamous cell carcinoma and adenocarcinoma. This lymphoma is closely associated with Epstein-Barr virus (EBV) infection and primarily affects nasal or upper aerodigestive structures, but can also extend to extranasal sites including skin, soft tissue, testes, and gastrointestinal tract. The hallmark feature of ENKTCL-NT is necrosis due to vascular damage caused by the tumor cells, involving angionecrosis and angiodestruction. It exhibits a distinct geographic and racial predilection, being more common in Asian and Latin American countries [1]. Diagnosing ENKTCL-NT is challenging, particularly in nonendemic regions and among children, as it can mimic reactive or inflammatory processes. The tumorigenesis of ENKL remains unclear, necessitating further studies to elucidate its molecular pathology. EBV is found in most cases of NK-cell leukemia/lymphoma, suggesting its oncogenic role, with patients potentially harboring biclonal or polyclonal malignant cell populations based on differential EBV genome incorporation [2].

### Case report

A 78-year-old male presented with crusted nasal lesions for approximately one year and progressive development of rhinomegaly (Fig. 3A e 3B). The patient reported being a former smoker. His past medical history included hypertension, inguinal hernia repair, and intestinal diverticulosis. He underwent an initial otolaryngological examination at another center. A first nasal mucosa biopsy showed non-diagnostic inflammatory material. Subsequently, it was decided to perform a second biopsy of granular tissue from the left nasal cavity and the mucosa of the right middle meatus. At the onset the patient’s performance status (PS) was assessed as 1.

#### Investigations

The complete blood count was within normal limits, with no peripheral lymphocytosis. Serum renal profile and liver function tests showed normal findings. The viral serology for EBV was positive (EBER-SISH test), while the viral serology for Human Immunodeficiency Virus (HIV), Hepatitis C Virus (HCV), and Hepatitis B Virus (HBV) was negative. The lactate dehydrogenase (LDH) level was within the normal range. The proliferation index was very high, as expected, with Ki67 at 90%. Computed tomography (CT) scan of the facial mass conducted prior to admission to our institution revealed dense material, measuring 5.9 cm, in the anterior and superior nasal cavities, adjacent to the nasal septum bilaterally, without evidence of contrast medium extravasation during the arterial phase, suggesting the absence of active bleeding. Additionally, soft tissue thickening was noted in the nasal alae, along with hypertrophy of the left turbinates. The magnetic resonance imaging was not performed due to the prolonged waiting times and the immediate necessity to start treatment. The histopathological examination of the lesion conducted on the second biopsy, both morphologically and immunohistochemically, indicated extranodal NK/T-cell lymphoma, nasal type, consistent with the WHO 2017 classification. The malignant lymphoid cells exhibited medium and large size, with eosinophilic cytoplasm and irregular nuclei containing granular chromatin. Additionally, numerous high endothelial venules were observed, along with focal areas of angioinvasion. Immunohistochemical analysis revealed that the malignant lymphoid cells tested positive for CD3, CD43, Granzyme B, and CD56, with some variability in expression. Regarding the perforation of the septum, it was noted that it was mentioned only in the biopsy report among the biopsied areas, but not in the imaging, where it was never actually observed. The images we were able to take showed no signs of cartilage or bone destruction, so we can assume that there was no destruction. Whole-body Positron Emission Tomography-CT (PET-CT) scan revealed pathological hyperfixation of the radiopharmaceutical in several regions: a notably extensive soft tissue mass within the nasal pyramid, with enlargement most pronounced in the area projecting towards the nares (SUVmax 37.1, metabolic volume 2.45 cc, polypoid tissue thickening at the level of the nasal septum, exophytically extending into the left nasal cavity (SUVmax 8.6) and a globular lymph node formation approximately 10 mm in size in the right lateral cervical area, level II (SUVmax 7.5). However, at the ultrasound the lymphnode was found to have a purely reactive nature. Using the Ann Arbor system the disease was classified as limited stage IE disease.

#### Treatment

A multidisciplinary board chose exclusive radiotherapy treatment with curative intent considering age (78 years old), PS (1), comorbidities and limited staging (IE).

#### Radiation therapy

**Fig. 1:**
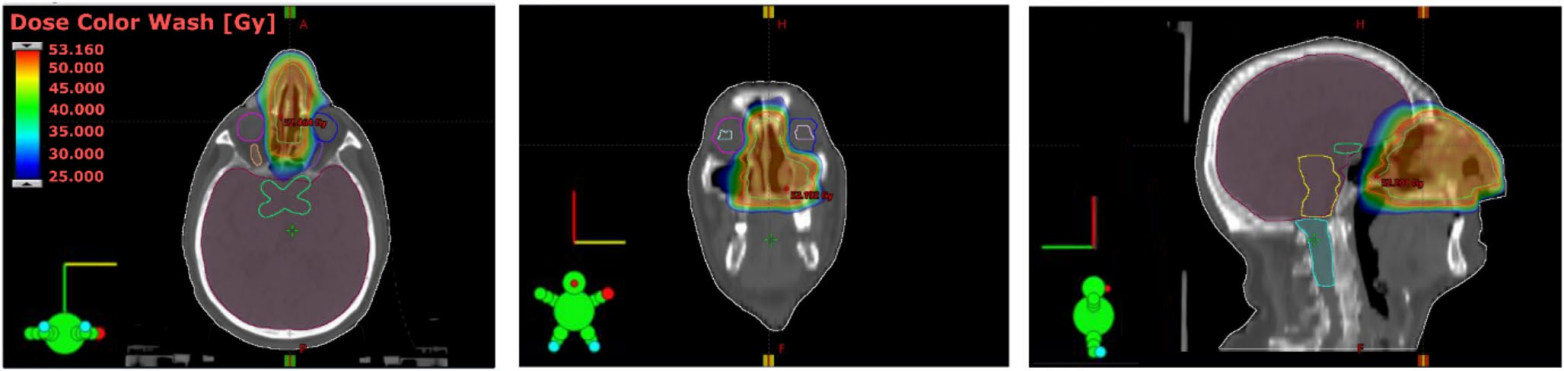
The distribution of doses (absolute doses)

**Fig. 2:**
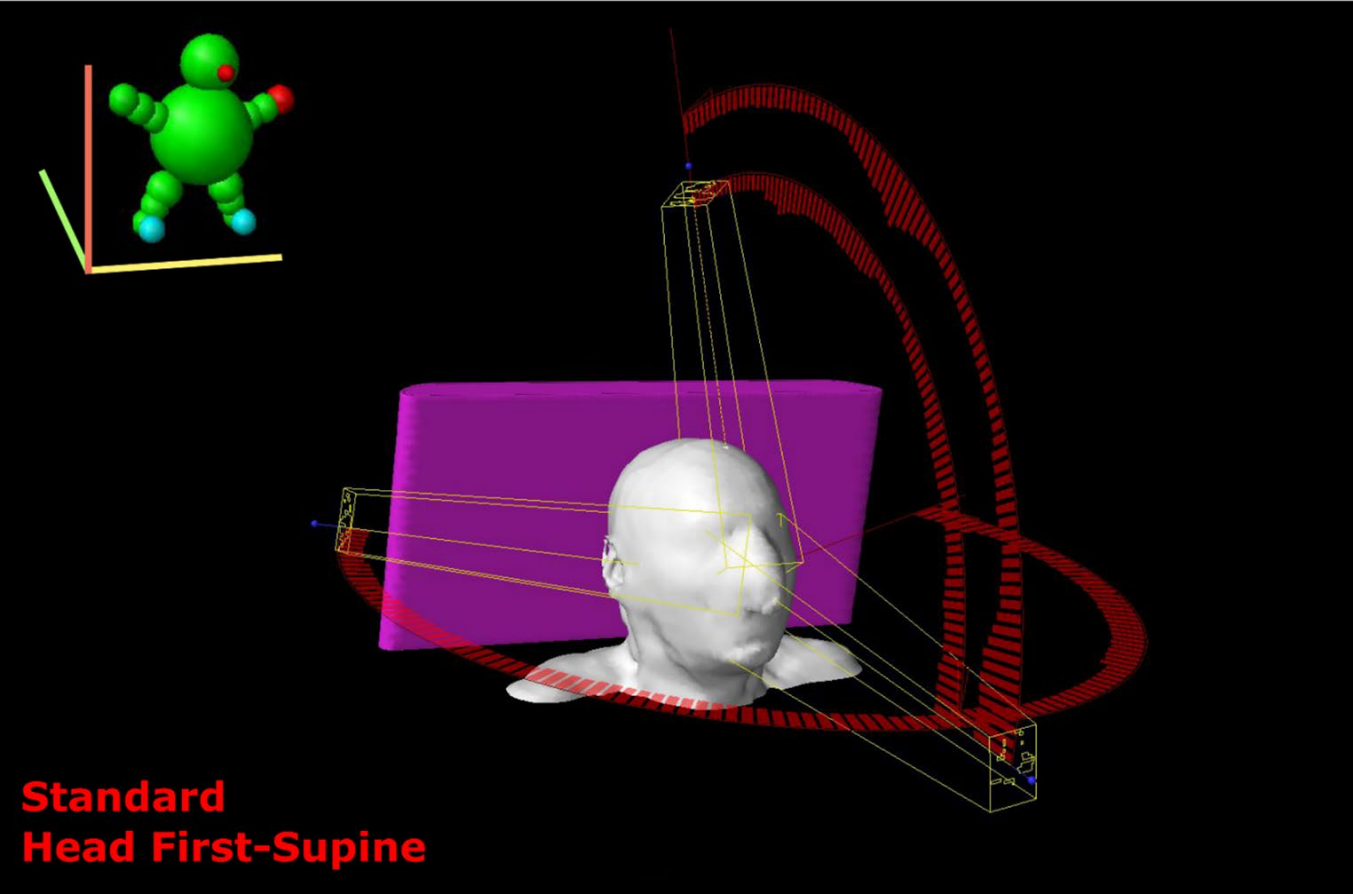
The arch geometry

The patient was immobilized as in head and neck cancer treatment, with a thermoplastic mask, in the supine position. He received CT simulation, taking care that the position was stable, reproducible, and comfortable during the CT simulation and irradiation. An image registration with PET scans was done to better define the disease. The GTV (Gross Tumor Volume) was designed as the sum of involvement found on clinical examination and imaging (CT, and PET/CT). The CTV (Clinical Target Volume) covered the GTV as well as potential sites of microscopic disease. According to Guidelines from the International Lymphoma Radiation Oncology Group for limited stage I nasal ENKTCL the CTV includes the high-risk structures based on the frequency of involvement and risk of relapse (whole nasal cavity, ipsilateral medial maxillary wall, anterior ethmoid sinuses, hard palate, posterior nasal aperture). The CTV expands further to fully cover the disease extension include the involved facial subcutaneous soft tissue with a bolus of 0.5 cm. The PTV (planning Target Volume) is an expansion of the CTV that accounts for setup uncertainties, in this case we used a margin of 0.4 cm from the CTV. The 6 MV photon Volumetric Modulated Arc Therapy (VMAT) plan was generated using the Eclipse treatment planning system, with calculations performed using the Acuros XB algorithm. The plan was delivered by a Varian True Beam linear accelerator equipped with a 120-leaf Millennium multi-leaf collimator. Three arcs were employed: two partial coplanar arcs at couch 270°, one clockwise from 0° to 120°, and the other counterclockwise from 120° to 0°, along with a partial non-coplanar arc at couch 0°, from 240° to 120°. This geometry optimizes the dose gradient outside the target region while sparing surrounding organs at risk. Specifically, the arc at couch 270° was crucial in minimizing dose to optical structures in close proximity to the target (Figs. 1 and 2). During the treatment a daily image guided setup with cone beam CT was done. The treatment was carried out with VMAT technique. We provided a total dose of 50 Gy in 25 fractions over 5 weeks (conventional fractionation). The OARs that we have considered were: parotid glands, brain stem, spinal cord, retina, optic nerves, optic chiasm, cornea, and lens. The recommended maximum dose constraints considered were: 54–50 Gy brain stem, 40 Gy spinal cord, 50 Gy optic nerve and chiasm, 45 Gy retina, 50 Gy cornea, and 10 Gy lens. A target adaptation was created twice for the tumor shrinkage during radiation therapy [3, 4].

#### Outcome and follow up

**Fig. 3:**
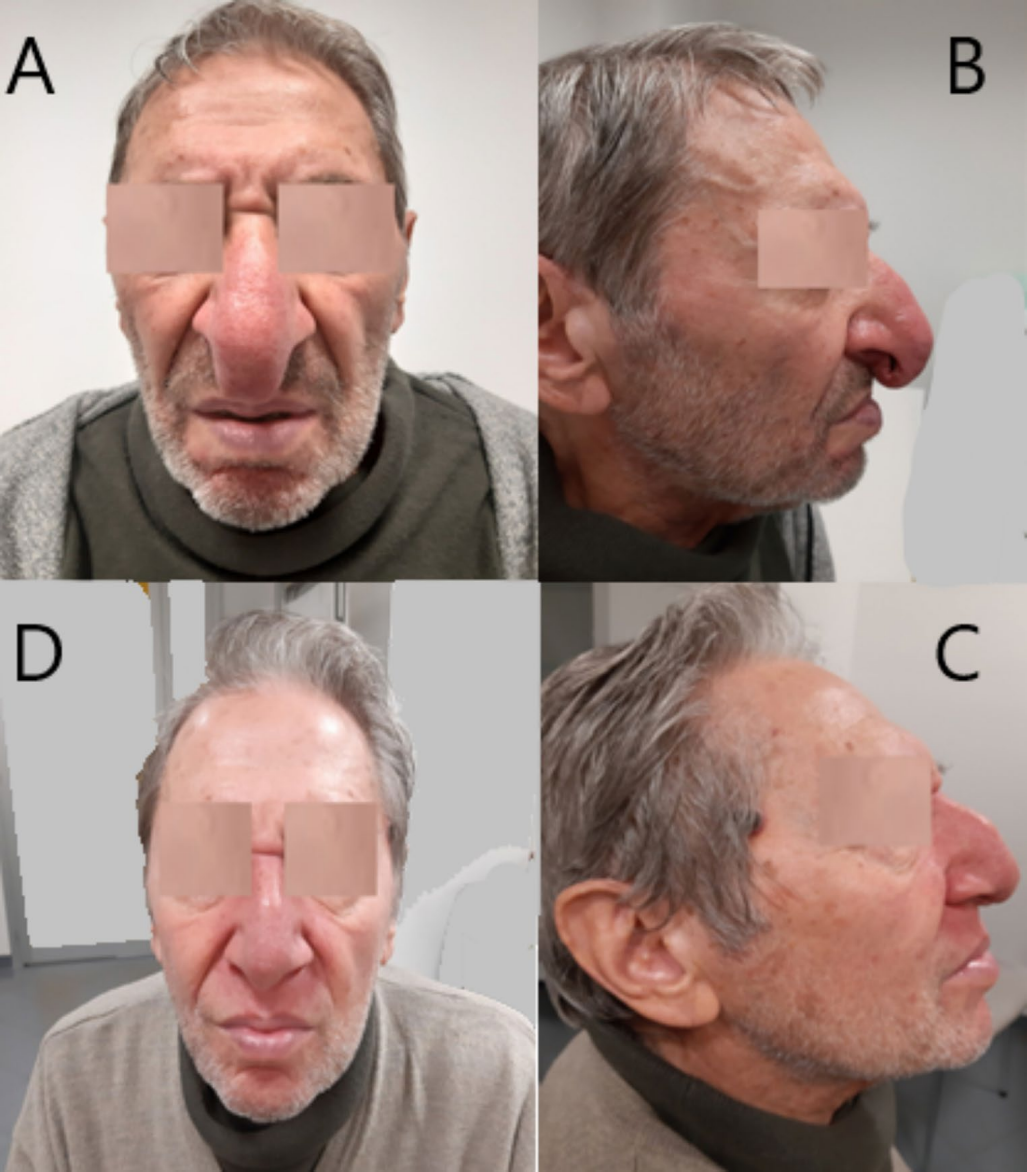
Rhinomegaly on the frontal (**A**) e lateral side (**B**) at baseline of radiotherapy. Nose as returned to on the frontal (**D**) and lateral side (**C**) one year after treatment

Radiation-induced toxicities were recorded and graded according to the Radiation Therapy Oncology Group and the European Organization for Research and Treatment of Cancer radiation morbidity scoring criteria. Based on the Revised Response Criteria of Malignant Lymphoma, tumor responses were assessed through physical examinations, nasopharyngoscopy and PET-CT. A comprehensive evaluation for the tumor response at 50 Gy, with fiber optic examination, documented a complete clinical response. The PET-CT scans at 3 and 6 months post-treatment show a progressive reduction in SUV values, ultimately reaching Score 2 according to 5-point Deauville Lymphoma Scale. In particular, there was no longer evidence of increased metabolic activity in the soft tissues of the nasal pyramid and septum, except for faint uptake at the tip of the nose (SUVmax 3.0 vs. SUVmax 37), along with a reduction in the size of the tissue volume in the left nasal cavity, with no increased metabolic activity observed. The patient is now one year post-treatment, in complete clinical (Fig. 3) and laboratory remission, with LDH levels within normal limits. A follow-up PET scan is scheduled shortly.

## Discussion

ENKTCL-NT has emerged as a distinct clinical entity, with significant advancements in understanding its clinical presentation, histology, genetics, and molecular characteristics. Over the past decade, substantial progress has been made in clarifying the disease’s heterogeneity, improving staging with advanced diagnostic imaging, and developing novel prognostic models and risk stratifications. Radiation therapy has traditionally been the main treatment modality for ENKTCL-NT. Historically, localized-stage ENKTCL was treated with large-field radiotherapy, sometimes combined with chemotherapy. However, recent studies have not confirmed the added benefit of doxorubicin-based chemotherapy for early-stage disease [5]. In this context, the study by Deng T et al. confirmed that, compared to RT alone, radiochemotherapy did not result in prolonged complete remission (CR), 5-year overall survival (OS), or 5-year progression-free survival (PFS), nor did it reduce severe flare-ups (SF) or local failures (LF) in patients with early-stage (IE/IIE) ENKTCL [6]. Modern Radiation Therapy for Extranodal Nasal-Type NK/T-cell Lymphoma provides risk-adapted therapy, target volume, and dose guidelines from the International Lymphoma Radiation Oncology Group [7]. Multidisciplinary management is crucial in the early stages for optimizing cure [8]. The primary pattern of spread is local disease invasion into adjacent structures. Regional lymph node involvement is uncommon and typically follows an orderly pattern. The precise definition and coverage of the target area, as well as the dose delivered, are critical, as several studies have shown improved locoregional control with optimal radiation parameters. There is a linear relationship between improved locoregional control and prolonged OS or PFS. For example, a Chinese retrospective study of 1332 patients with localized ENKTCL demonstrated a dose-dependent association of radiation therapy with locoregional control, progression-free survival, and overall survival [9, 10]. In another Chinese study (CLCG study), a radiation dose ≥ 50 Gy significantly decreased the risk of locoregional recurrence, with a 5-year cumulative locoregional failure rate of 37.5% for patients receiving a dose < 50 Gy, versus 8.8% for those receiving ≥ 50 Gy (*p* = 0.0028). Wei et al. showed that a dose ranging from 48 to 56 Gy resulted in an 80% complete response rate, with a low local relapse rate (8.6%) within the radiation field. Kaplan-Meier analysis indicated that RT dose was a significant predictor of OS and disease-free survival (*p* < 0.05), and high doses (≥ 54 Gy) independently predicted a better prognosis [11, 12]. Advances in imaging and conformal radiation therapy techniques have improved outcomes while reducing the side effects of radiation therapy. Intensity-modulated radiation therapy (IMRT), specifically VMAT in our case, ensures excellent target coverage and dose conformity, while sparing normal tissues [13, 14]. There is currently limited evidence regarding the use of particle therapy. However, based on experiences in head and neck cancer and lymphoma, proton therapy may offer benefits in terms of local control and reduced toxicity [15]. Acute toxicities are typically transient, dose-dependent, and confined to the irradiated volume, while late toxicities and secondary malignancies are rarely reported [3]. Advances in imaging and conformal radiation therapy methods have significantly improved outcomes, minimizing side effects. For intermediate or high-risk patients (e.g., tumor invasion, elevated LDH, stage II, Eastern Cooperative Oncology Group score 2, or age > 60), combined-modality treatment is recommended. This includes radiation therapy followed by non-anthracycline-based chemotherapy, brief chemotherapy followed by radiation, or concurrent chemoradiation. asparaginase-based regimens, such as SMILE (dexamethasone, methotrexate, ifosfamide, L-asparaginase, etoposide), GELOX, P-GELOX (pegaspargase, gemcitabine, oxaliplatin), and AspaMetDex (L-asparaginase, methotrexate, dexamethasone), are considered standard treatments. However, due to the poor prognosis (5-year OS of 10-40%), there is a critical need for novel treatment strategies, and prospective clinical trials are strongly encouraged [16].

## Conclusions

Significant advancements have been made in the understanding and management of extranodal NK/T-cell lymphoma, nasal type over the past two decades. Improved diagnostic techniques, prognostic models, and treatment strategies have led to improved survival outcomes, particularly in early-stage disease. Radiation therapy remains a crucial component of the treatment regimen for ENKTCL-NT [16]. The optimal dose and target volume for radiation therapy continue to be refined, with studies demonstrating a clear dose-response relationship for locoregional control and survival. Advances in radiation therapy techniques, such intensity-modulated radiation therapy (IMRT) and volumetric modulated arc therapy (VMAT), have improved target coverage and reduced toxicity, further enhancing treatment outcomes. Continued research and development are essential to further improve outcomes for patients with ENKTCL-NT and address the unmet needs of this patient population.

## Data Availability

No datasets were generated or analysed during the current study.
